# Upscaling the Fabrication of Rod‐Shaped Microgels: A Microfluidic Method Combining Step‐Emulsification With Consecutive Droplet Confinement in Parallelized Microchannels

**DOI:** 10.1002/adma.74822

**Published:** 2026-08-28

**Authors:** Matthias Mork, Ninon Möhl, Greta Romahn, Laura Klasen, Cédric Bergerbit, Laura De Laporte

**Affiliations:** ^1^ Institute for Technical and Macromolecular Chemistry (ITMC) Chair of Macromolecular Materials for Medicine RWTH Aachen University Aachen Germany; ^2^ DWI—Leibniz Institute for Interactive Materials e. V. Aachen Germany; ^3^ Department of Advanced Materials For Biomedicine (AMB) CBMS—Center For Biohybrid Medical Systems AME—Institute of Applied Medical Engineering University Hospital RWTH Aachen Aachen Germany

**Keywords:** anisotropy, chip, fabrication, materials science, microfluidics, microporous material, nanotechnology, particle, polymerization, taxonomy

## Abstract

Anisometric rod‐shaped microgels are an emerging material class holding potential for tissue engineering. Their anisotropic shape has proven advantageous in the fabrication of granular hydrogels and microporous annealed particle scaffolds (MAPs), featuring larger pore sizes in comparison to their spherical counterparts. However, to enable the use of rod‐shaped microgels as building blocks for high‐throughput tissue models, a robust and scalable production method is needed. Here, we report a microfluidic fabrication method to produce rod‐shaped microgels by combining step‐emulsification (SE) and droplet confinement. We highlight the development of the microfluidic chip design, and characterize the properties of the microgel rods produced via on‐chip gelation, using two light‐induced polymerization chemistries. Compared to single‐channel microfluidic techniques, rod‐shaped microgels are generated in eight parallelized microchannels in a relevant size range for tissue engineering applications, holding potential for further upscaling.

## Graphical abstract

A microfluidic method is presented which enables parallelized fabrication of rod‐shaped microgels, based on step‐emulsification (SE) droplet generation and subsequent droplet distribution and confinement, combined in a single device. Microgel rods are fabricated in parallel at different flow rates based on two light‐mediated crosslinking chemistries, and their potential as promising building blocks for tissue engineering and biofabrication applications is discussed.

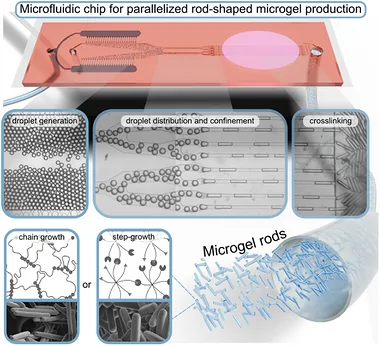

## 1 Introduction

Tissue engineering demands material systems that can closely mimic the properties of native tissues. In this regard, hydrogels have been extensively studied in the last decades. Despite their capability to create environments similar to the native extracellular matrix (ECM) [1, 2], hydrogels typically feature an isotropic network with nanometer‐scale mesh sizes, impeding cell‐cell interaction, cell migration, and cell proliferation throughout the material, which all rely on the ability of the hydrogel to degrade [3]. Promising hydrogel alternatives in tissue engineering have emerged in the form of granular hydrogels. Granular hydrogels are three‐dimensional (3D) structures composed of individual micrometer‐scaled building blocks like microgels, which, upon interparticle crosslinking, are classified as microporous annealed particle scaffolds (MAPs) [4, 5]. Their primary advantage over bulk hydrogels is that they feature pore sizes in the micrometer scale, enhancing the initial cell infiltration and overall cell–cell interaction within the material. At the same time, the microgel network supports the diffusion of oxygen and nutrients. So far, most granular hydrogels are composed of spherical microgels, whereas recent studies have shown the potential of using rod‐shaped microgels [5, 6, 7, 8, 9]. Notably, MAPs composed of spherical microgels offered an interstitial space, ranging from 10–45 µm depending on their initial diameter [4], while employing anisometric building blocks revealed pore sizes up to several hundred of micrometers and provided an increased scaffold porosity [5, 10]. Furthermore, it has been shown that rod‐shaped microgels can also be employed as injectable building blocks for granular hydrogels through physical jamming, providing enhanced porosities in comparison to spherical microgels and facilitating cell infiltration [6, 9]. Apart from providing scaffolds with larger porosity, rod‐shaped microgels can introduce controllable directionality into a material [11, 12, 13], which is especially relevant for structured tissues, including the spinal cord, cardiac tissue, or muscle [14]. The creation of large pore sizes, increased scaffold porosity, directionality, and injectability provided by rod‐shaped microgels highlight their relevance in the generation of native‐like 3D tissue models.

Biocompatible anisometric microgels can be produced either with batch or continuous processes. Batch production methods include, particle replication in nonwetting templates (PRINT), based on soft‐lithographic micromolding [15], or photolithography approaches [8]. These methods facilitate particle production with defined shapes over wide size ranges but represent noncontinuous processes with low yields, decreasing their relevance for applications with high material demand. In contrast, continuous processes such as microfluidics allow for on‐demand adjustment of production parameters and offer a high potential for upscaling, making them well‐suited for the large‐scale production of anisometric microgels.

In microfluidics, rod‐shaped microgels are typically produced by droplet elongation via confinement coupled with controlled and rapid crosslinking mechanisms, using triggers like light [5, 6, 16, 17], temperature [18], or a change in pH [19]. Typically, the length of the resulting microgels can be controlled by adjusting the flow rate ratio of the dispersed (*Q_disp._*) and continuous (*Q_cont._*) phase, while the width of the droplets is predefined by the width of the microchannel. An exception is compartmentalized jet polymerization (CJP), as it enables the continuous generation of rod‐shaped microgels featuring widths that are significantly smaller than the microchannel dimensions [20].

The majority of existing microfluidic systems producing rod‐shaped microgels with relevant properties and feature sizes for tissue engineering are considered low in throughput, as they are operated at precursor flow rates below 0.2 mL/h [5, 17, 19, 20]. One reason for this is the intended anisometric microgel geometry, requiring a minimum residence time on‐chip to maintain the elongated shape after crosslinking. Further, the flow within the microfluidic channels has to be retained within regimes that prevent extensive fluctuations or a change of the flow profile. Exemplarily, in a recent publication, microgel rods have been fabricated with a single‐channel microfluidic device, revealing a change of the global microgel rod geometry due to flow perturbations at higher applied precursor flow rates, indicating a reduced fabrication robustness [9]. In contrast to rod‐shaped microgels, the upscaling of spherical particles has been extensively investigated, yielding production rates in the range of a few mL/h up to L/h, depending on the production technique and materials [21, 22, 23, 24, 25]. In microfluidic systems, upscaling typically involves the parallelization of individual microfluidic channels. Besides producing parallelized microfluidic masters via common photolithographic approaches, rapid prototyping has been previously investigated in fabricating parallelized 3D microfluidic devices [26]. Apart from the parallelization of flow‐focusing channels (FF) [23, 24], parallelized step‐emulsification (SE) has emerged as a promising microfluidic technique in the high‐throughput fabrication of spherical droplets and particles [27, 28, 29, 30, 31]. The concept of the SE method relies on the microfluidic device configuration, featuring an array of individual shallow nozzle channels that are connected to a deeper reservoir channel. During operation, the sudden increase in channel height induces an expansion of the dispersed phase into the deeper channel, resulting in the formation of microdroplets, while being less flow rate dependent than FF devices [27]. To date, SE has been intensively used for the generation of spherical droplets or particles but also more complex geometries, like double emulsions, enabled through droplet reinjection into a second SE device [29, 32]. Despite these advances, limited progress has been made to parallelize the production of anisometric microgels to achieve similar production capacities as for spherical particles. Recently, a silicon‐based microfluidic device for the large‐scale production of microgels was reported, which uses parallelized FF channels [33]. While initial results with this device have demonstrated that rod‐shaped microgels can be produced via UV polymerization of a poly (ethylene glycol) diacrylate (PEG‐DA) pre‐polymer solution, specific production parameters and limitations were not systematically defined [33]. Moreover, the FF method is more sensitive in terms of dispersed phase and continuous phase flow rate, since the generation of the droplets and their geometry is highly dependent on the balance of these flow rates and corresponding flow profile [34]. In addition, the employed silicon‐based microfluidic devices are more expensive than polydimethylsiloxane (PDMS) microfluidic devices, and the preparation requires specific equipment.

To address the low production rates in fabricating rod‐shaped microgels, we developed a microfluidic method, combining SE and droplet confinement using a single chip. The method consists of three steps: (1) parallelized generation of spherical droplets using SE, (2) droplet distribution and confinement into parallelized narrowing microchannels, and (3) UV‐triggered on‐chip photo polymerization (Figure 1). Following to the droplet generation, an efficient and balanced droplet distribution into the narrowing microchannels is required to enable robust droplet elongation and crosslinking. The droplet distribution was achieved through the introduction of hierarchical bifurcations of the main channel. Using two‐photon polymerization (2PP) 3D printing, a master structure was fabricated. The reusable master‐mold enables the reproducible production of PDMS microfluidic devices via soft‐lithography, featuring distinct height profiles and inclined height transitions. The parallelization of individual microchannels divides the total flow rate among multiple channels, reducing the flow velocity in each channel and thereby increasing the on‑chip residence time. Therefore, higher production rates can be achieved for fabricating biocompatible rod‐shaped microgels in a relevant size range for tissue engineering applications (Table S1). Rod‐shaped microgels were produced at polymer precursor flow rates up to 3 mL/h, using different polymer compositions and photo‐polymerization chemistries. Specifically, using free radical polymerization (FRP) with PEG‐DA 700 and radical‐mediated thiol‐ene click chemistry, based on a mixture of star‐PEG‐norbornene (sPEG‐NB) and degradable star‐PEG‐ester‐thiol (sPEG‐ester‐SH), we compared the influence of the crosslinking chemistry on the required pre‐polymer concentration to fabricate stable microgel rods. In addition, the influence of the applied UV dose on the mechanical properties of the resulting microgels was assessed. To assess the potential of our obtained microgel rods in various tissue engineering applications, we also investigated their injectability through extrusion experiments and their ability to support myogenic differentiation and the formation of myosin heavy chain using C2C12 mouse myoblasts.

**FIGURE 1:**
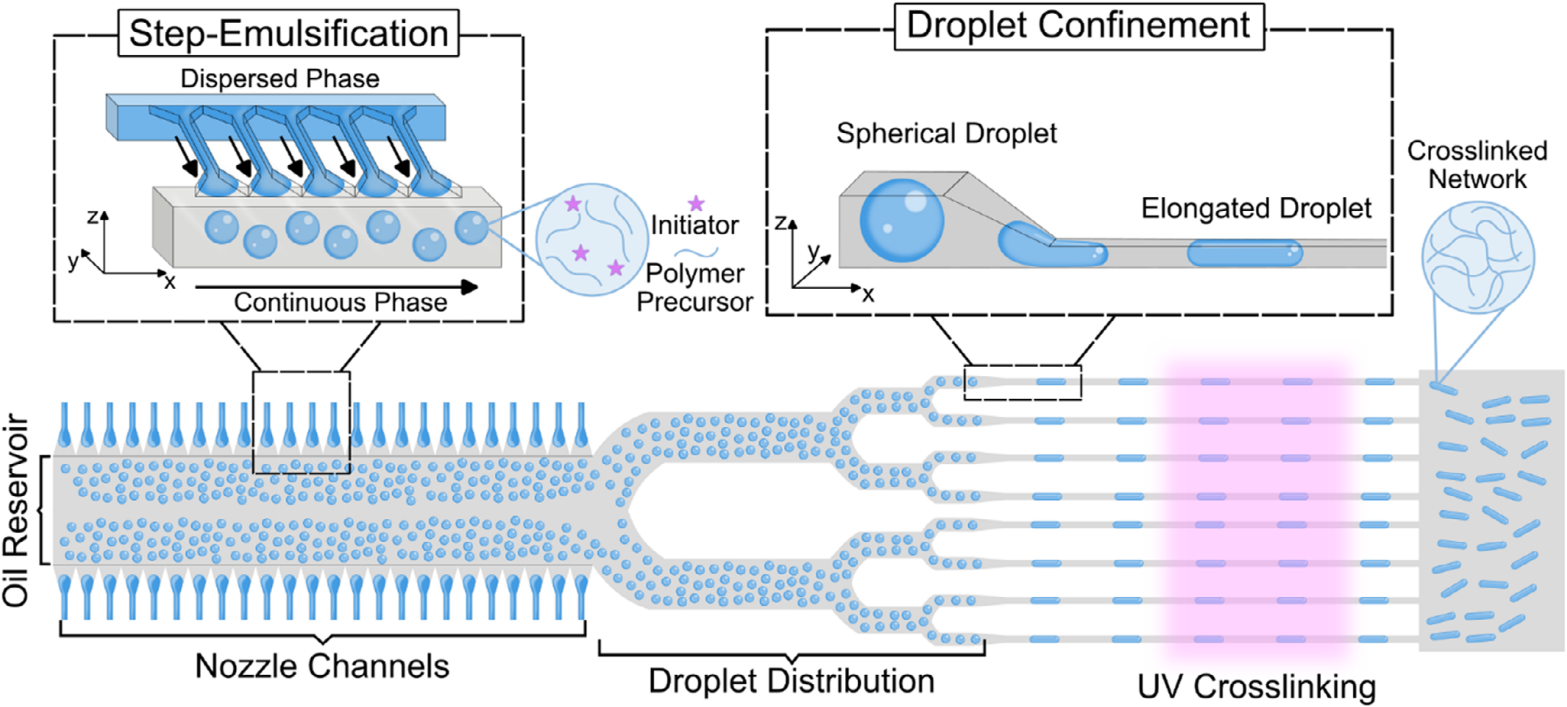
Schematic overview of the microfluidic method to parallelize the fabrication of rod‐shaped microgels. Spherical droplets are generated in a SE section. In SE, the dispersed phase is distributed into multiple parallel nozzle channels and droplet formation is achieved by expansion of the dispersed phase into a deep oil reservoir. After droplet formation, the droplets are distributed by gradually splitting the main channel and confined in an array of parallelized microchannels. UV light is used to trigger a crosslinking reaction on‐chip to generate rod‐shaped microgels.

In summary, this work demonstrates a technique to upscale the production of anisometric microgels for applications with larger material demand, such as bioprinting, automated high‐throughput production of in vitro tissue models, or the regeneration of larger tissue defects in the body [6].

## 2 Results and Discussion

### 2.1 Development of the Microfluidic Chip Design

To produce rod‐shaped microgels in an array of parallelized microchannels, we fabricated microfluidic masters using 2PP 3D printing. Compared to photolithography approaches, 3D microfabrication of master structures allows for seamless integration of complex height profiles. Compared to previously demonstrated layer‐by‐layer rapid prototyping approaches [26], 2PP 3D achieves significantly higher resolutions, enabling a facile fabrication of microfluidic channels below 100 µm widths [35]. In the context of this work, height profiles are not only relevant for the droplet generation via SE but also for subsequent droplet confinement and elongation in an array of parallelized microchannels. In this regard, we fabricated two microfluidic master prototype designs with different geometries (Figure S1) used for the fabrication of PDMS microfluidic devices. One particularly investigated design feature was the presence or absence of a droplet distribution section prior to droplet elongation. Based on the observations that we made with the prototype designs (Section S2), such as uneven droplet distribution and flow velocity, we next fabricated an optimized microfluidic master design, with all characteristic dimensions of the microfluidic chip design displayed in Figure S2. In this microfluidic channel, the dispersed phase enters the oil reservoir from two opposing sides through the nozzle channels, as displayed in Figure 2A. This channel configuration, inspired by previously published SE designs [36], facilitates increasing the number of nozzle channels, without compromising extensive widening of the main channel, which could impede droplet formation and distribution. In total, the SE section featured 48 nozzle channels. We gradually split the main channel a total of three times, to distribute the spherical droplets into eight parallel microchannels with a cross‐sectional area of 50 × 50 µm^2^. The functionality of the microfluidic device was evaluated by using an aqueous solution composed of liquid PEG‐DA 700 (30% (w/w)) (Figure 2B). To test the limits of the optimized microfluidic chip design, we investigated the droplet generation, distribution, and confinement for three different flow rate combinations, measuring the initial spherical droplet diameter and the length of the elongated droplets within the confinement microchannels (Figures 2C and S3; Movie S1). Initially, the flow rate ratio was fixed at *Q_disp_:Q_cont_* equal to 1:2. For the lowest flow rate combination at *Q_disp. :_ Q_cont._* = 1:2 mL/h we measured a diameter of approximately 102.32 ± 4.63 µm, which increased to 116.07 ± 5.46 µm at *Q_disp. : _Q_cont._* = 2:4 mL/h, and to 126.69 ± 6.96 µm at *Q_disp. :_ Q_cont._* = 3:6 mL/h. Although the droplet size in SE is reported [27] for being less sensitive to variations in flow rate in comparison to FF microfluidic devices, our measurements reveal that the droplet diameter is increasing with increasing flow rates. This could be an indication that at the higher flow rates, the device is starting to transition from a capillary force‐driven regime toward a regime where viscous forces become significant [37]. We hypothesize that the increase in droplet diameter might be attributed to an increased backpressure caused by droplet confinement.

**FIGURE 2:**
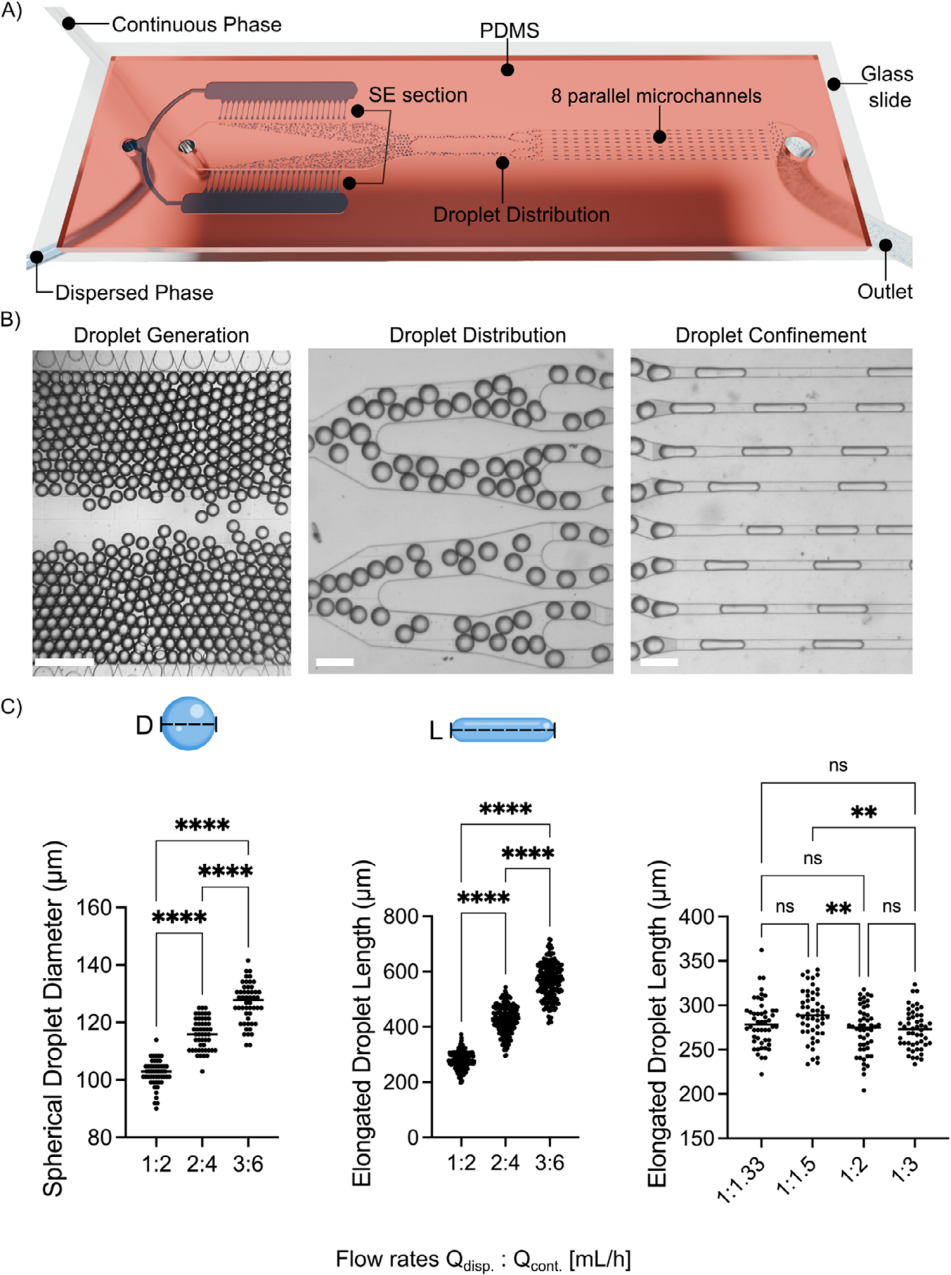
Overview of the microfluidic chip design during operation. (A) Schematic of the microfluidic PDMS device, highlighting the different sections. (B) Brightfield image series showing (from left to right) droplet generation in the SE section, droplet distribution, and confinement of the droplets. Scale bar in left image depicts 500 µm, all other scale bars represent 200 µm. (C) Influence of the applied flow rates on the initial spherical droplet diameter (D) (left) and the length (L) of the elongated droplet within the confinement microchannel (right). *n* = 50 spherical droplets, *n* ≥ 50‐200 elongated droplets. Statistical significance was determined by one‐way ANOVA with Tukey's multiple comparison test (*p < 0.05; **p < 0.01; ***p < 0.001; ****p < 0.0001).

As expected, the droplet size increase was also reflected in the measured lengths of the elongated droplets after confinement. Here, we obtained for each flow rate combination droplet lengths of 278.07 ± 31.25 µm, 427.54 ± 50.39 µm, and 565.80 ± 63.44 µm, respectively. Further, we observed larger deviations in droplet length within each population, compared to the diameter of the spherical droplets. While in spherical droplets, minor volume changes result in slight deviations of the droplet diameter, the same volume changes are more pronounced for the length of an elongated droplet. The reason for this is that the droplet is confined within a constant channel width and height and can exclusively extend along the channel length. Variations in the length are, therefore, reduced when calculating the volume, which is the same volume of the spherical droplet, calculated using the power of three of the original diameters. Hence, it was expected that the elongated droplets would feature a broader length distribution, according to the size variation of the initial microdroplets. Here, the low and medium flow rate ratios are considered to be more optimal as the observed droplet length variation was less than for the highest tested flow rate. Apart from the total applied flow rates, we further investigated the influence of the flow rate ratio on the elongated droplet length (Figures 2C and S3). Maintaining a constant *Q_disp._* = 1 mL/h, we varied the continuous flow rate, *Q_cont_*_,_ from 1.33, 1.50, 2.00, and 3.00 mL/h, respectively, and measured the resulting droplet lengths, indicating that the elongated droplet diameter is independent of the flow rate balance, which is in accordance with observations in conventional SE devices [27, 30]. Generally, we expect that by parallelizing a larger number of nozzle channels and confinement microchannels, the absolute numbers of the maximum stable flow rates can be further increased, while maintaining comparable droplet dispersities, indicating the scalability of the method. Based on our observations, we considered *Q_disp. _* and* Q_cont._* = 3 and 6 mL/h to be the maximum achievable flow rate combination of the present device, with regard to generating and elongating spherical precursor droplets. Based on the individual droplet volume and total processed volume of the polymer per hour (flow rate dispersed phase), we calculated the estimated amount of droplets produced per hour to be approx. 1.78*10^6^ per hour (flow rate dispersed phase 1 mL/h), 2.44*10^6^ per hour (flow rate dispersed phase 2 mL/h), and 2.82*10^6^ per hour (flow rate dispersed phase 3 mL/h). It is important to note that the increase is not linear due to the increasing spherical diameter with increasing flow rate. To give rise of further droplet diameters and elongated droplet lengths, we additionally explored microfluidic masters with an adjusted nozzle geometry (Figure S4), which as previously demonstrated [27, 30], is a key parameter for influencing the resulting microdroplet diameter. We observed a significant decrease in droplet diameters (up to >40%) and resulting reduced elongated lengths, depending on the nozzle height, with further details provided in the Section S3.

After establishing the fundamental steps regarding droplet generation, distribution, and confinement, we focused on investigating the on‐chip photo‐polymerization of the elongated droplets to fabricate rod‐shaped microgels using FRP and radical‐mediated thiol‐ene click chemistry.

### 2.2 Fabrication of Nondegradable PEG‐DA Rod‐Shaped Microgels Based on a FRP

To fabricate rod‐shaped microgels, we operated the microfluidic device with a pre‐polymer solution supplemented with a photoinitiator, enabling the crosslinking of the precursor droplets in their elongated state. As previously reported [20], an elongated droplet has to be subjected to a certain minimum UV dose to ensure sufficient crosslinking for maintaining the elongated rod shape, which can be either controlled by residence time or UV intensity. Parallelizing microgel rod production spreads the total flow rate among several channels, thus each rod receives a higher UV dose without increasing the overall intensity. Consequently, higher total flow rates can be achieved in parallelized channels while matching the UV dose of a single channel. Using 30% (w/w) PEG‐DA 700 Da supplemented with 1% (w/w) of lithium phenyl‐2,4,6‐trimethylbenzoylphosphinate (LAP) photoinitiator, undergoing a free‐radical polymerization (Figure 3A), we investigated the minimum UV dose required to produce stable rod‐shaped microgels at *Q_disp. : _Q_cont._* = 1:2 mL/h in our parallelized set‐up (Figure 3B). We initiated the crosslinking using a UV diode, featuring a circular spot with a diameter of approximately 6 mm in combination with a UV‐resistant photomask on the channel, to ensure a uniform irradiation length of approximately 5.5 mm (Figure S5).

**FIGURE 3:**
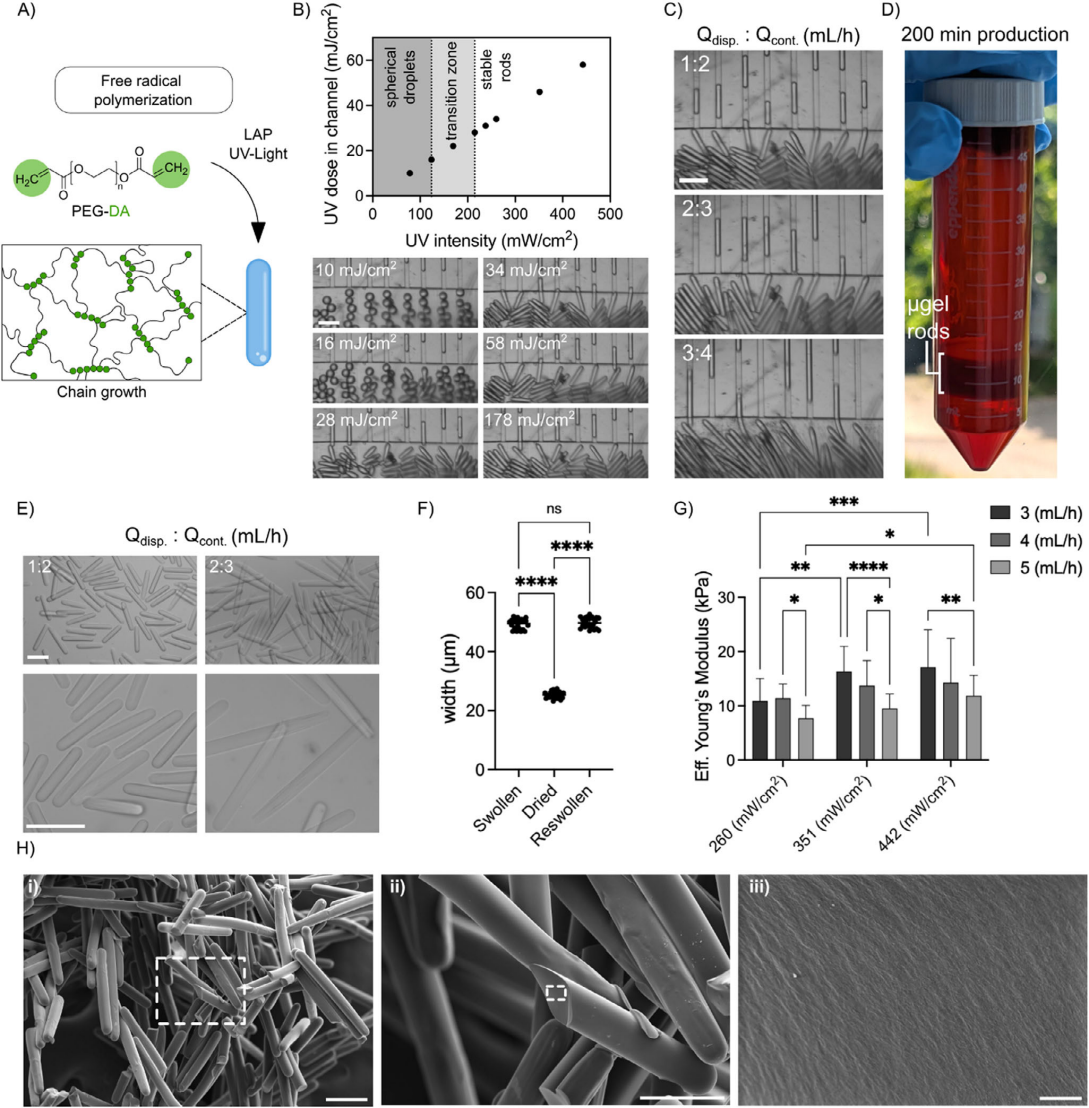
Photo‐crosslinking elongated PEG‐DA droplets into rod‐shaped microgels in our parallelized microfluidic device. (A) Chemical structure of PEG‐DA undergoing a FRP during photo‐crosslinking, initiated by LAP photoinitiator, resulting in a chain‐growth polymer network. (B) Analysis of the applied UV dose required to fabricate stable microgel‐rods (30% (w/w) PEG‐DA 700) at a total flow rate of 3 mL/h. Microscope images show the droplet morphology upon subjecting them to the respective UV dose. The scale bar = 200 µm and is representative for all images in this series. (C) Microscope images showing microgel rod production at three different total flow rates. UV crosslinking was performed at comparable UV doses of 46–53 mJ/cm^2^. Scale bars = 250 µm. (D) Visual representation of the produced microgel rods in a 50 mL falcon tube, fabricated over a total time of 200 min at flow rates of *Q_cont._* = 2 mL/h and *Q_disp._* = 1 mL/h. (E) Exemplary microscope images of collected microgel rods after purification at two different flow rate combinations, irradiated at the same UV dose (46 mJ/cm^2^). Scale bars = 200 µm. (F) Swelling behavior of rod microgels produced at a total flow rate of 3 mL/h and 351 mW/cm^2^. (G) Mechanical properties of rod‐shaped microgels produced at different total flow rates and UV doses, obtained via nanoindentation at different total flow rates and UV doses. Statistical significance was determined by one‐way ANOVA with Tukey's multiple comparison test (*p < 0.05; **p < 0.01; ***p < 0.001; ****p < 0.0001). (H) Scanning electron microscopy (SEM) images of rod microgels produced at 351 mW/cm^2^ and a total flow rate of 3 mL/h. The square displays the magnified area on the images from left to right. Scale bar = 100 µm (i), 50 µm (ii), 1 µm (i).

Experimentally, at UV doses below 16 mJ/cm^2^, the precursor droplets remained mostly spherical. For higher UV doses up to around 31 mJ/cm^2^, we observed partial crosslinking of the droplets. We, therefore, considered 31 mJ/cm^2^ as the minimum required UV dose for stable microgel‐rods fabrication. For higher applied UV doses, no further visual changes in the microgel shape were observed. Furthermore, we theoretically investigated the interdependence of flow velocity and UV dose, by calculating the UV dose in our parallelized device and plotted this against the total applied flow rate (*Q_total_*) (Figure S5). We compared the values to a single‐channel FF device, featuring the same microchannel cross‐section diameter of 50 × 50 µm^2^. The calculations revealed that for a 30% (w/w) PEG‐DA solution, our parallelized microfluidic device would provide a sufficient UV dose for total flow rates of over 9 mL/h. In comparison, the single‐channel microfluidic device only achieves the critical UV dose at total flow rates below 2 mL/h. Even after considering the smaller rectangular spot size of irradiation with a length of ∼5.5 mm in our parallelized microchannels instead of the 6.0 mm diameter circular spot in the single channel, the applied UV dose, and in turn the total achievable flow rate, is expected to be 7.5 times larger by parallelizing eight channels instead of a single channel. This demonstrates the opportunity of the microfluidic device to be used as a large‐scale platform with significantly higher flow rates than previously reported, while maintaining homogeneous material properties. We highlighted this by operating the microfluidic chip pre‐polymer flow rates (*Q_disp_*) up to 3 mL/h and different total flow rates (*Q_tot_*) up to 7 mL/h, resulting in crosslinked rod‐shaped microgels (Figure 3C). To demonstrate the robustness of the microfluidic device, we continuously fabricated microgel rods for at least 200 min, as can be seen in Movie S2 and Figure 3D. Moreover, we assessed the integrity of the microfluidic channel before and after continuous production, revealing no significant alteration caused by the microgel fabrication (Figure S6). We additionally investigated the total flow rate limit for a reduced PEG‐DA 700 concentration of 15% (w/w), which was determined to be below 4.5 mL/h in our parallelized device. This is attributed to the higher minimum UV dose limit (118 mJ/cm^2^) needed to crosslink the microgels on chip due to the reduced polymer concentration (Figure S7), demonstrating the importance of rapid crosslinking kinetics, which has to be considered for further upscaling the throughput of an individual microchannel. With regards to tissue engineering applications, we did not further increase the initiator concentration to avoid increasing amounts of free radicals. Furthermore, in previous research, an increase in initiator did not necessarily increase the crosslinking rate, as this concentration reaches a plateau quickly [17].

We further characterized microgel‐rods with 30% (w/w) PEG‐DA 700, fabricated at different total flow rates. After purification, the microgels featured comparable widths of approximately 50 µm, while the lengths and aspect ratios (AR) varied depending on the total flow rates (Figure 3E), which is in accordance to the droplet size increase at higher flow rates, highlighted in Figure 2. We further characterized the microgels by analyzing their swelling behavior (Figure 3F) and the mechanical properties of the microgels via nanoindentation (Figure 3G). As depicted in the graph, the microgels collapse to about half of their swollen diameter when dried, after which they fully reswell. We tested different combinations of the total applied flow rate and the set UV intensity to characterize the variability of microgel stiffness. The lowest obtained stiffness was 7.8 ± 2.1 kPa at a UV intensity of 260 mW/cm^2^ at *Q_total_* = 5 mL/h, representing the lowest analyzed UV dose. Consequently, the highest stiffness with 16.8 ± 6.7 kPa was obtained at *Q_total_* = 3 mL/h and a UV intensity of 442 mW/cm^2^, representing the highest investigated UV dose. Overall, the mechanical data demonstrates that the stiffness of the microgels can be altered to a certain degree by either changing the UV intensity or the total flow rates. In turn, these results reveal that comparable mechanical properties can be achieved by adjusting the system to similar UV doses by changing the UV intensity, independent of the applied flow rates. We also looked at the surface morphology of the obtained rod microgels after purification through scanning electron microscopy (SEM) (Figure 3H). The images, displaying the microgels in dried state, show a relatively smooth surface without a visible open pore structure (Figure 3Hiii).

### 2.3 Fabrication of Degradable sPEG‐norbornene/sPEG‐ester‐thiol Rod‐shaped Microgels via Thiol‐ene Click Chemistry

An alternative, faster crosslinking chemistry was employed to ensure efficient crosslinking at elevated flow rates, thereby enabling higher production rates and greater flexibility in the selection of polymer concentrations. The norbornene‐thiol chemistry also allows for the use of degradable thiol‐containing crosslinkers, containing esters or matrix metalloprotease (MMP)‐sensitive peptide domains. FRP is known to be sensitive to oxygen inhibition, making it challenging to increase the production rate without increasing the polymer or initiator concentration. Oxygen acts as a radical scavenger in FRP to form peroxy radicals exhibiting a lower reactivity that results in less chain growth. However, in tissue engineering applications, reducing the polymeric material and the number of free radicals is favorable. Light‐mediated thiol‐ene click chemistry using norbornene and thiol is well known in the literature [38] due to its reactivity and compatibility for a wide range of polymers [38]. This type of crosslinking follows a step‐growth mechanism that is less affected by oxygen inhibition. The reason for that is the easy abstraction ability of the thiol hydrogen [38, 39]. Furthermore, step‐growth mechanisms offer greater control over the network formation and typically present more homogeneous networks compared to FRP. Microgels have been produced with this chemistry in single‐channel devices using polymers including hyaluronic acid (HA) and PEG [40, 41]. As reactants, we used 8‐arm star PEG‐ester‐thiol (sPEG‐SH, 20 kDa) and 8‐arm sPEG‐norbornene (sPEG‐NB, 20 kDa), keeping the amount of initiator constant at 1% (w/w) (Figure 4A). Introducing an ester moiety to the network enables the degradation of the rod microgels hydrolytically [42, 43], further increasing their functionality. Additionally, norbornenes are electron‐rich olefins, providing fast reaction kinetics, furthermore supported by the ring strain of the structure [38, 44, 45].

**FIGURE 4:**
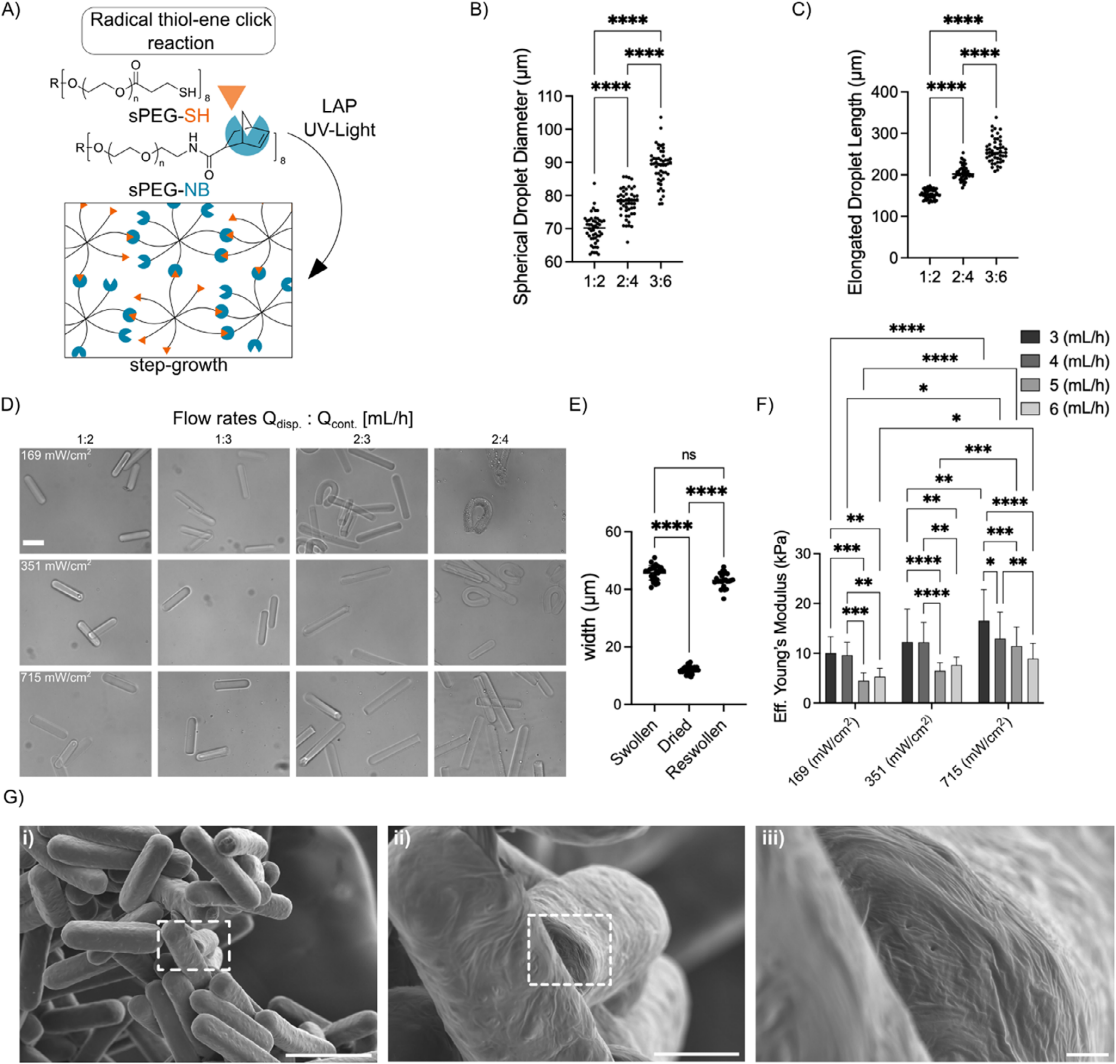
Photo‐crosslinking elongated 5% (w/v) PEG‐norbornene and PEG‐ester‐SH droplets into rod‐shaped microgels in our parallelized microfluidic device. (A) Chemical structure of PEG‐norbornene and PEG‐ester‐SH undergoing light‐mediated thiol‐ene click crosslinking, initiated by LAP photoinitiator, resulting in a step‐growth polymer network. (B+C) Influence of the applied flow rates on the initial spherical droplet diameter (B) and the length (C) of the elongated droplet within the confinement microchannel. n = 50 spherical droplets and elongated droplets. Statistical significance was determined by one‐way ANOVA with Tukey's multiple comparison test (*p < 0.05; **p < 0.01; ***p < 0.001; ****p < 0.0001). (D) Microscope images of the purified rods dispersed in water with their respective production parameters. Scale bar = 100 µm. (E) Swelling behavior of rod microgels produced at a total flow rate of 3 mL/h and 351 mW/cm^2^. (F) Mechanical properties of rod‐shaped microgels produced at different total flow rates and UV doses, obtained via nanoindentation at different total flow rates and UV doses. Statistical significance was determined by one‐way ANOVA with Tukey's multiple comparison test (*p < 0.05; **p < 0.01; ***p < 0.001; ****p < 0.0001). (G) Scanning electron microscopy (SEM) images of rod microgels produced at 351 mW/cm^2^ and a total flow rate of 3 mL/h. The square displays the magnified area on the images from left to right. Scale bar = 50 µm (i), 10 µm (ii), 1 µm (i).

We first screened the reactivity of our precursor solutions, analyzing bulk hydrogels using rotational rheology (Figure S8). The total PEG concentration was set to 3 and 5% (w/v), where a lower polymer concentration results in a higher gelation time (2.81 ± 1.4 s and 0.96 ± 0.65 s, respectively). We then compared the thiol‐ene bulk hydrogels to PEG‐DA hydrogels consisting of 5%, 15%, 30%, and 40% (w/w) (Figure S8). For the PEG‐DA, the gelation times were similar to the norbornene‐thiol chemistry (between 0.6 and 2 s), except for 5% (w/w) PEG‐DA, where an increase to 12.61 ± 0.56 s was observed. Furthermore, the thiol‐ene samples demonstrated storage moduli (G’) of 4.00 ± 0.038 kPa and 12.90 ± 0.40 kPa for the 3 and 5% (w/v) PEG hydrogels, respectively, which is in a similar range as the 15 and 30% (w/w) PEG‐DA hydrogels, which had storage moduli of 7.20 ± 0.40 kPa and 18.30 ± 1.90 kPa, respectively, even though the thiol‐ene samples comprise much lower polymer concentrations. This is most likely due to the differences in chain length and polymer architecture, which have been demonstrated to affect the crosslinking density. For example, PEG‐DA leads to softer hydrogels, compared to star PEG‐acrylate at similar polymer concentrations, which also influenced microfluidic rod production [17, 46]. Furthermore, the differences in chain growth (FRP) and step growth (thiol‐ene click) may lead to differences in mechanical properties. We again investigated the droplet generation, distribution, and confinement for three different flow rate combinations, measuring the initial spherical droplet diameter and the length of the elongated droplets within the confinement microchannels (Figure 4B,C) using 5% (w/v) of sPEG‐norbornene/sPEG‐ester‐SH. Similar to PEG‐DA, we observe an increase in droplet size and elongated droplet length with increasing flow rate. However, the droplet size of sPEG‐SH/sPEG‐NB microgel rods is significantly smaller (smallest rod length PEG‐DA 278.07 ± 31.25 µm *vs* smallest rod length sPEG‐SH/sPEG‐NB 153.50 ± 11.37 µm), indicating the impact of the precursor solution on the rod size. The robustness of sPEG‐SH/sPEG‐NB microgel production was again demonstrated by continuously producing microgel rods at for a total time of at least 100 min (Movie S3).

We probed four different flow rates alongside four different UV intensities (Figures 4D and S9) to determine the boundaries at which microfluidics settings we would obtain partially stable rods (bent shape) or stable rods (straight shape). Here, we observed that with higher flow rates, higher UV intensities were required to produce stable rods, which was also confirmed after purification (Figure 4D). Compared to PEG‐DA, the lowest UV intensity to obtain stable rod microgels was at 22 mJ/cm^2^ instead of 31 mJ/cm^2^ when using a flow rate of 3 mL/h (Movie S4). This minimum intensity shifted with higher flow rates, as is shown in the brightfield images of the produced rod microgels (Figure 4D). The successful fabrication of rod‐shaped microgels from a 3% (w/v) precursor solution demonstrates that the presented approach is applicable at different polymer concentrations (Figure S10). As no qualitative differences in device operation were observed, the remaining experiments were conducted with the 5% (w/v) formulation, which served as the standard system for detailed characterization. Similar to the microgels obtained using PEG‐DA, the microgels collapse to about half of their initial swollen width (Figure 4E). Furthermore, the stiffness increases with increasing UV intensity and decreasing flow rates. The lowest Eff. Young's modulus with 4.50 ± 1.60 kPa was obtained at an intensity and flow rate of 169 mW/cm^2^ and 5 mL/h, while the highest modulus of 16.50 ± 6.30 kPa was observed at 715 mW/cm^2^ and 3 mL/h (Figure 4F). At higher flow rates, such as 5 and 6 mL/h, some rods had a bent shape due to the lower UV dose. This is in line with the low recorded Young's moduli. To obtain straight rods, 351 mW/cm^2^ at 5 mL/h and 715 mW/cm^2^ at 6 mL/h would be required. Similar to the PEG‐DA rods, we investigated the surface morphology of the sPEG‐NB/SH rods using SEM (Figure 4G). Interestingly, the sPEG‐NB/SH rods exhibited a very different surface roughness than the PEG‐DA rods. The higher magnifications even exposed small open network structures (Figure 4Giii). This difference is most likely due to the much lower polymer concentration and the different reaction mechanism.

### 2.4 Demonstrating the Potential of the Microgel Rods for Tissue Engineering Applications

The potential of employing microgels (spheres and rods) in 3D (bio)printing applications has been shown in previous publications [47, 48]. The low production rate was previously identified [6] as a bottleneck for using microgel rods in such applications, requiring larger microgel quantities. We believe that our microfluidic device can facilitate the application of microgel rods in this context by enabling parallelized and scalable microgel rod production. For this purpose, we exemplarily investigated the extrudability of two types of microgel rods, fabricated using both of the presented chemistries (FRP with PEG‐DA and thiol‐ene click with sPEG‐SH/NB). We first jammed the microgels via centrifugation before loading them into the syringe, followed by manual extrusion through a 27G needle. In the case of the PEG‐DA microgel rods, the extrusion resulted in a continuous microgel rod filament, without clumping (Figure 5A). As can be seen from Figure 5A, the majority of the microgels within the extruded filament appear to be aligned in the direction of the extrusion, which is in line with previous observations [48]. The extrusion of sPEG‐NB/SH microgel rods occasionally resulted in filament discontinuities, localized clump formation, and reduced filament homogeneity under otherwise identical extrusion conditions (Figure 5B). This behavior may be attributed to the enhanced microgel aggregation observed following centrifugation. In direct comparison to PEG‐DA, the sPEG‐NB/SH microgel rods showed no clear alignment parallel to the extrusion flow (Figure 5B). The differences in these two behaviors can most likely be attributed to the different pre‐polymer solutions. Employing sPEG pre‐polymers compared to linear PEG‐DA might give rise to physical interactions between the dangling uncrosslinked polymer chains, enhancing microgel aggregation in close microgel‐microgel contact. In the future, further analysis of varying extrusion parameters will be required to determine how the distinct microgel rod characteristics (e.g. material, length, stiffness) influence the printability, clumping, and structural alignment of the microgel rods after extrusion. Nevertheless, by fabricating two extrudable microgel rod types in larger quantities, the findings demonstrate the potential of this novel microfluidic method to generate (bio)printing‐compatible microgel rods, which can be further tailored to future tissue‐specific requirements.

**FIGURE 5:**
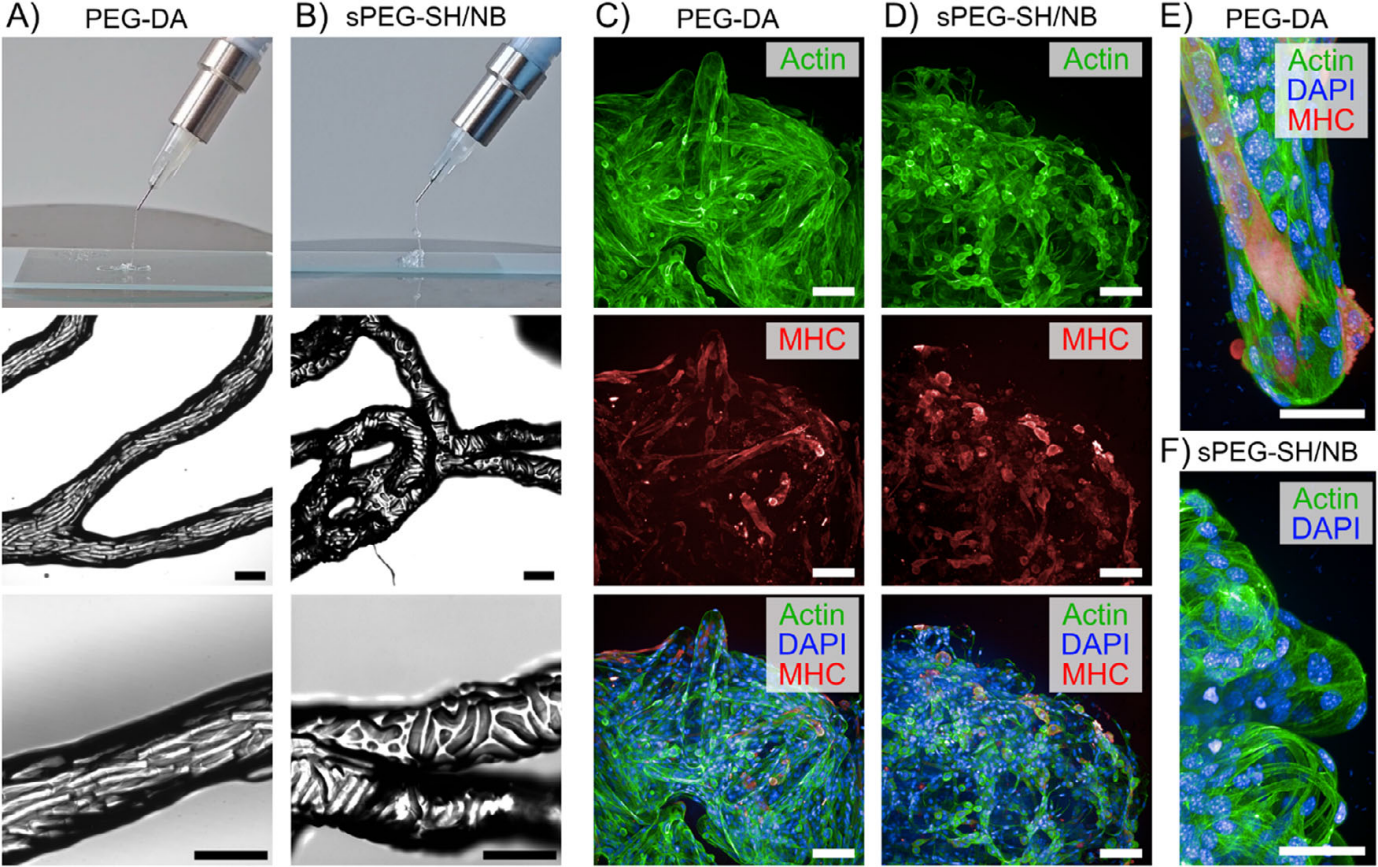
Tissue engineering applications—Needle extrusion experiments and cell material interaction. (A) Assessment of the extrudability of 30% (w/w) PEG‐DA microgel rods using a 27G needle (top). Brightfield h of the extruded 3D filament (middle) and magnified image (bottom) with scale bars =250 µm. (B) Extrusion experiment of 5% (w/v) sPEG‐SH/NB microgel rods through a 27G needle (top). Corresponding brightfield image of the extruded filament (middle) and magnified image (bottom). The scale bars = 250 µm. (C–F) Cell material interaction using C2C12 mouse muscle cells (500 cells/microgel) on GRGDS‐PC‐functionalized rod microgels employing the different chemistries. The cells cultured in the scaffolds were differentiated for a total time of 7 days. (C) Cell‐assembled scaffold with 30% (w/w) PEG‐DA microgel rods with DAPI (blue) and actin (green) and expression of myosin heavy chain (MHC) (red). Scale bars= 100 µm. (D) Cell‐assembled scaffold with 5% (w/v) sPEG‐SH/NB microgel rods with DAPI (blue) and actin (green) and expression of MHC (red) within the scaffold. Scale bar= 100 µm. (E) Representative image of a multinucleated myotube on the surface of a PEG‐DA microgel rod. The scale bar = 50 µm. (F) Magnified image showing the arrangement of the actin filaments on the sPEG‐SH/NB microgel rods. The scale bar = 50 µm.

Additionally, to further show the potential of our microfluidic device and the obtained microgel rods, we performed initial cell experiments assessing the cell/material interactions and cell compatibility using C2C12 mouse myoblasts. We functionalized both microgel types with GRGDS‐PC to promote integrin‐mediated cell adhesion and cultured the C2C12 mouse myoblasts together with the microgels to form a scaffold and culture the cells for 7 days. We subsequently assessed the myogenic differentiation and cytoskeletal organization through staining for myosin heavy chain and actin, with the results shown in Figure 5C–F. With both microgel types, we observed myosin heavy chain, indicating that both types are compatible with C2C12 survival and differentiation (Figure 5C,D). In contrast, samples containing cells alone showed little or no detectable myosin heavy chain expression (Figure S11). Therefore, the microgel rods provide a more favorable myogenic environment than cell‐only control conditions. Microgel rods without GRGDS‐PC biofunctionalization did not demonstrate cell‐material interaction and were therefore not incorporated into the C2C12 clusters (Figure S11). This indicated that integrin‐mediated cell adhesion is crucial for the myogenic differentiation‐promoting effect of both microgel types. However, clear differences were observed in the extent and the organization of myotube formation between the two rod types. PEG‐DA rods (Figure 5C,E) supported more pronounced myotube formation than the sPEG‐NB/SH rods (Figure 5D). Multinucleated myotube‐like structures were particularly observed using PEG‐DA rods (Figure 5E). These findings suggest not only a promotion of myosin heavy chain expression when employing PEG‐DA rods, but also the morphological maturation and fusion of differentiating myoblasts. The smoother surface observed for PEG‐DA rods (Figure 3H) compared to sPEG‐NB/SH rods (Figure 4H) and the greater length may contribute to this effect. Longer rods could provide a more continuous anisotropic cue, allowing the cells to align over a greater distance before fusion, thus facilitating the formation of elongated, multinucleated myotubes. Furthermore, the actin cytoskeleton appeared highly aligned with the orientation of these rods, whereas the sPEG‐NB/SH samples exhibit a less aligned and more diffusely distributed actin network (Figure 5F). This also corresponds with less extensive myotube formation. Both microgel types differ in length and surface topography, as well as in the underlying formed network, which are potential causes of the observed cellular response. In summary, both microgel types supported myogenic differentiation of C2C12 myoblasts, demonstrating cell compatibility and potential benefits for future tissue engineering applications.

## 3 Conclusion

For the first time, a SE‐based microfluidic approach was employed to parallelize the fabrication of rod‐shaped microgels. The presented platform enables the robust production of rod‐shaped microgels with tunable dimensions and material properties while significantly increasing production throughput compared to conventional serial approaches. Its versatility was demonstrated by implementing two different crosslinking chemistries, highlighting the flexibility of the platform to accommodate materials with distinct crosslinking kinetics and functional properties. Furthermore, the injectability of the fabricated microgels and their interaction with C2C12 cells demonstrate the potential of the produced rod‐shaped microgels as promising building blocks for tissue engineering and biofabrication applications.

The presented platform represents an important step toward the scalable manufacturing of anisotropic microgels for applications requiring larger material quantities, such as automated liquid handling and 3D bioprinting. While the current device already achieves a substantial increase in production rate, further upscaling through increased parallelization of both SE nozzles and confinement channels remains an important direction for future work. In addition, we envision the development of a modular “plug‐and‐play” library of interchangeable droplet‐generation and confinement sections with varying geometries, enabling the straightforward production of rod‐shaped microgels with smaller widths, different AR, and tailored dimensions while maintaining robust and scalable operation.

## 4 Experimental Section/Methods

### 4.1 Chemicals

Quinoline yellow (Sigma Aldrich, 95%), oil red o (Sigma Aldrich), toluene (Alfa Aesar, 99.7%), lithium phenyl‐2,4,6‐trimethylbenzoylphosphinate (LAP, Sigma Aldrich, ≥95%), poly(ethylene glycol) diacrylate average Mn 700 (PEG‐DA, Sigma Aldrich), Sylgard 184 silicone elastomer kit (The Dow Chemical Company), 3 M Novec HFE 7500 (Ionic liquids Technologies, >99%), Krytox 157 FSH (DuPont), n‐hexane (Fisher Scientific, ≥99%), Span 80 (Sigma Aldrich), propan‐2‐ol (IPA, Fisher Chemical, ≥99.8%), trichloro‐(1H,1H,2H,2H‐perfluorooctyl)‐silane (Sigma Aldrich, 97%), L‐ascorbic acid (Sigma Aldrich, 99%), propylene glycol monomethyl ether acetate (PGMEA, Sigma Aldrich, ≥99.5%), 8 arm poly (ethylene glycol) norbornene Mn 20 kDa (>95%, CreativePegworks) were used as received. MilliQ water was used for the preparation and purification of the microgels.

### 4.2 Microfluidic Master Mold Fabrication

The same procedure was applied for the fabrication of all different microfluidic master structures presented in this work. In short, a negative 3D structure of the microfluidic channel geometry was designed in AutoCAD 2024, processed in DeScribe 2.7, and was fabricated using two‐photon direct laser writing (Nanoscribe GmbH, Photonic Professional GT). As previously described [35], an acryl‐silanized glass coverslip (LABSOLUTE Th. Geyer 76 × 50 × 1 mm) was covered with a photoresist (Nanoscribe, IP‐S) and mounted in the 3D printer. A 25x objective was mounted in the printer and the structure was fabricated with a blocksize of X:200, Y:200, Z:160 µm at 15° shear. After printing completion, the master structure was submerged in PGMEA for 10 min to remove uncured polymer resin, followed by washing with IPA solution for 3 min. The substrate was dried under nitrogen flow and post‐cured for 4 h using UV light (200 mW, λ = 365 nm). The cured structure was fluoro‐silanized with trichloro‐(1H,1H,2H,2H‐perfluorooctyl)‐silane (50 µL per glass coverslip) in a desiccator under high vacuum overnight.

### 4.3 Microfluidic Chip Fabrication

The microfluidic chips were fabricated as previously described [49], with minor variations. In short, PDMS base and curing agent (10:1 (w/w)) were mixed with a dye solution (100:1 (w/w)). The dye solution was prepared by mixing oil red O (60 mg, 3.00% (w/w)) and quinoline yellow (3 mg, 0.15% (w/w)) followed by dissolving in toluene (2 g). The PDMS mixture was cast on the master structure, degassed, and cured at 60°C for 12 h. The PDMS replica was cut from the master mold and inlet and outlet holes were punched (inlet: 0.75 mm, outlet: 1.2 mm biopsy puncher, Electron Microscopy Science). The PDMS slab and a glass slide (Marienfeld 75 × 50 × 0.13 mm) were washed sequentially with IPA and deionized water, followed by drying under nitrogen flow, before plasma treatment and bonding. The microfluidic chip was stored at 60°C for 12 h to complete the bonding. The device was rendered hydrophobic by chemical vapor deposition (CVD) with trichloro‐(1H,1H,2H,2H‐perfluorooctyl)‐silane (50 µL per chip) in a vacuum desiccator overnight. Afterwards, the outside of the chip was rinsed with Novec 7500 and wiped using lint‐free tissues.

### 4.4 Synthesis of m‐arm poly (Ethylene Glycol) Ester Thiol

8‐arm PEG‐ester‐SH was prepared by Fischer esterification of the respective PEG‐(OH)_m_ according to the literature [50]. Briefly, in a 250 mL two‐neck round bottom equipped with a Dean‐Stark PEG‐(OH)_m_ (20000 Da sPEG‐OH 8 arm) (1 eq., 1.00 g) was dissolved together with 3‐mercaptopropionic acid (40 eq.) and *p*‐toluenesulfonic acid *(p*‐TsOH) (0.1 eq.) in 70 mL cyclohexane. The reaction was refluxed at 130°C for 24 h, and at least two volumes of Dean‐Stark were removed. Afterwards, the reaction mixture was cooled down to r.t. and the cyclohexane was separated from the product via decantation. The remaining white wax was dissolved in DCM (50 mL) and washed with saturated NaHCO_3_ (2 × 15 mL), brine (1 × 15 mL) and dried over MgSO_4_. The solid is removed by filtration and the solution is concentrated until a viscous oil is obtained. The resulting oil is dissolved again in minimal amount of DCM (2‐3 mL) and precipitated by dropwise addition in excess cold (−20°C) diethyl ether (40x vol DCM) and the final product is isolated as a white powder (isolated yield 70%). 1H NMR (CDCl3, 400 MHz): δ (ppm) = 4.26 (4H, m, –CH2CH2OC(O)–), 3.64 (133H, br), (CH2CH2O)n), 2.77 (4H, m, –CH2CH2SH), 2.68 (4H, m, –CH2CH2SH), 1.68 (2H, t, SH).

### 4.5 Microgel Production

Rod‐shaped microgels were prepared from aqueous solutions composed of PEGDA (15 or 30% (w/w)) or PEG‐norbornene/‐ester‐SH (5% w/v). LAP (1% (w/w)) was used as photoinitiator. The solutions were stored in brown glass vials until use, to protect from undesired light exposure. The continuous oil phase was composed of Novec HFE 7500 with Krytox 157 FSH surfactant (4.5% v/v) for PEG‐DA and FluoSurf‐C surfactant (0.5 wt%) for PEG‐norbornene/‐ester‐SH. The pre‐polymer and oil solutions were transferred into a syringe (Hamilton Gastight Series 1000, 5 mL) and the syringe containing the dispersed phase was wrapped in aluminum foil. In the case of PEG‐norbornene and PEG‐ester‐SH, both precursors were combined in one syringe. A 25G cannula was inserted into tubing (PTFE, OD 0.9 mm, ID 0.4 mm, TECHLAB GmbH) and attached to the syringes. Residual air was removed and the syringes were fixed in a syringe pump (Harvard Apparatus Pump 11 Elite). The other ends of the tubes, as well as an outlet tube (PE, OD 0.048 in, ID 0.03 in, Instech Laboratories) were inserted into the dedicated holes on the microfluidic device. The microfluidic chip was positioned on a custom made microfluidic chip holder with the glass side facing upwards. Microfluidic production was initiated by starting the flow of the continuous phase to prefill the collection reservoir. Afterwards, the dispersed phase was started until droplet formation was visible. The flow rates were adjusted as indicated. The microfluidic channel was covered with a UV resistant photomask, creating a rectangular irradiation field. A custom build UV diode [5] was positioned on top of the irradiation field and operated at different UV intensities, as indicated. To prevent unwanted radicals during the collection of the rod microgels, L‐ascorbic acid was added in excess to ethanol as a collection media for the PEG‐norbornene/‐ester‐SH system. Purification of the microgels was achieved by multiple subsequent washing steps, as previously reported [51]. In short, the excess continuous phase was pipetted off and the microgels were sequentially washed with Novec HFE 7500, n‐hexane + span 80 (1% v/v), n‐hexane, IPA, and Milli‐Q water. Each solvent was applied five times before proceeding to the next solvent.

### 4.6 Rheological Measurements of Bulk Hydrogels

Rheological characterization was carried out using a Discovery HR‐3 hybrid rheometer (TA Instruments, New Castle, DE, USA) with 20‐mm parallel plate geometry and a 50 µm gap. We transferred 381 µL of the pre‐polymer solution to the rheometer at 25°C followed by the time‐dependent measurement of polymerization under 3 mW cm^−2^ UV light with a frequency of 1 Hz and an oscillation strain of 1%. We also took frequency‐dependent measurements at a set oscillation strain of 1% and strain‐dependent measurements at a set frequency of 1 Hz. Each material was measured as triplicate samples.

### 4.7 Swelling Behavior

The purified microgels were analyzed in their swollen state first and then in their collapsed state through the addition of isopropanol by measuring their width. We subsequently added water to reswell and measured again. In total, 25 microgels were measured.

### 4.8 Nanoindentation

Before nanoindentation, the rod microgels were immobilized on a cell culture well plate using a poly‐L‐lysine solution (0.01% (w/v), Sigma‐Aldrich). The well surface was covered with a thin film of the solution and left overnight to evaporate. The microgels were subsequently pipetted on the surface of the well plate and left for sedimentation.

The mechanical properties of the rod microgels were assessed using a Pavone Nanoindenter (Optics11Life, Amsterdam, Netherlands). The Nanoindenter was equipped with a cantilever probe comprising a spherical tip radius of 3 µm and a cantilever stiffness of 0.510 N m^−1^. For each sample at least three microgels were indented. The piezo speed was set to 30 µm s^−1^ and the indentation depth to 0.3 µm. All measurements were conducted at r.t. and in water. The effective Young's modulus (Pa) was determined from the obtained load‐indentation curves employing the Hertz contact model. The data analysis was carried out using the Software Dataviewer V2.5 (Optics11Life).

### 4.9 SEM

Samples were fixed in 3% glutaraldehyde (Agar Scientific, Wetzlar, Germany) for at least 1 h at room temperature and stored at +4°C. They were then washed for 15 min with 0.1 M Sørensen's phosphate buffer (0.2 M, pH 7.39; Merck, Darmstadt, Germany). Dehydration was carried out in a graded ethanol series: 10 min each in 30%, 50%, 70%, and 90% ethanol, followed by three 10 min steps in 100% ethanol. The samples were subsequently subjected to critical point drying using liquid CO_2_ in a Critical Point Dryer (Polaron, Quorum Technologies Ltd, Ashford, Kent, UK). Dried specimens were sputter‐coated with a 10 nm layer of gold–palladium using a Sputter Coater EM SCD500 (Leica, Wetzlar, Germany) and examined in a scanning electron microscope (Quattro S Environmental SEM, Thermo Fisher Scientific, Eindhoven, The Netherlands; funded by the German Research Foundation, DFG project no. 495328185) at 10 kV under high vacuum.

### 4.10 Microgel Extrusion Experiments

The extrusion experiments were performed with both microgel rod types fabricated at flow rates of *Q_disp. :_ Q_cont._* = 1:2 mL/h at a UV intensity of 351 mW/cm^2^. The microgels were suspended in DI water and centrifuged for 5 min at 4000 rpm in a 5 mL Lobind Eppendorf tube. The supernatant was removed, and the microgel pellet was transferred into a glass Hamilton syringe using a positive displacement pipette. The remaining interstitial excess water was expelled by gentle pushing through a 30G ¼ inch straight nozzle. The microgels were then extruded by hand onto a microscope glass slide through a 27G ¼ inch straight nozzle. The microscope images were recorded on a Leica DMIL LED Fluorescence with 4X and 10X objectives.

### 4.11 Cell Experiments With C2C12 Muscle Cells

C2C12 mouse myoblasts (Sigma‐Aldrich) were cultured in growth culture medium containing Dulbecco's Modified Eagle Medium (DMEM, (Gibco 41965‐039)) supplemented with 10% fetal bovine serum (FBS Advanced) and 1% antimycotic‐antibiotic (AMB). Cells were incubated at 37°C with 5% CO2 before being used for further experiments. Myoblasts up to passage 7 were used for experiments. C2C12 cells were cultured in a T75 Flask until 80% confluency and dissociated using Trypsin (TrypLE Express (cell lab stock, Gibco, 12604)) for 5 min at 37°C. Afterward, cell number and viability were assessed; cells with viability between 90% and 95% were used in the experiments.

### 4.12 Microgel Coating and Preparation

Microgel rods were sterilized for 2 h under UV exposure. Afterward, the microgels were centrifuged at 2000 rpm (Eppendorf centrifuge 5810R) to generate a microgel pellet. From this pellet, 50 µL were transferred into protein‐low‐bind 500 µL Eppendorf tubes and resuspended in 100 µL PBS. 250 µg GRGDS‐PC peptide was added to biofunctionalize the microgels. The microgels were incubated in the coating solution overnight at 4°C. Afterward, the microgels were centrifuged into a pellet, and the supernatant was removed, followed by a resuspension in cell medium (DMEM + 10% FBS + 1% AMB).

### 4.13 Cell Experiment

In each well of a low‐attachment 96‐well plate, around 40 microgel rods were pipetted. Afterward, 20.000 cells were added in each well to reach a concentration of around 500 cells/microgel. Cell medium was added to each well to reach a final volume of 150 µL per well. The experiment was prepared on D0 using myoblast growth medium consisting of DMEM, 10% FBS, and 1% AMB. After experiment preparation, the well plate was centrifuged at 300 rcf for 5 min and subsequently placed in the incubator. On D1, the media was changed to differentiation media consisting of DMEM, 1% Horse Serum (Sigma‐Aldrich H1270), and 1% AMB. On D3, the media was exchanged with fresh differentiation media. In total, the cells were cultured with/without the microgels for 7 days.

### 4.14 Fixation and Immunohistochemistry

Samples were fixed with 4% PFA for 45 min, washed with 1 × PBS (pH 7.4) three times, and stored at 4°C in PBS or used directly for staining. The samples were incubated in a solution containing 4% BSA (SEQENS) and 0.1% Triton X‐100 (Sigma‐Aldrich) for 1 h to permeabilize the cell membrane and block. Before the staining procedure, samples were washed two times with PBS. Staining for Myosin heavy chain was performed with the MHC4 primary antibody (Myosin 4 monoclonal antibody (MF20) (eBioscience, 14‐6503‐82)) at a concentration of 1:200 overnight at 4°C. Afterward, samples were washed 2 times with PBS and incubated with the secondary antibody Alexa FluorTM 647 (1:200) and Phalloidin 488 (1:500) in PBS (Phalloidin‐iFluor 488 Reagent, abcam, (ab176753)). Afterward, samples were incubated with DAPI stain (1:200 in PBS) for 10 min (RT) and washed 2 times with PBS. Samples were stored in 1x PBS at 4°C prior to imaging. All confocal images were recorded using the Opera Phoenix Confocal microscope (revvity).

### 4.15 Statistical Analysis

Data points are means ± standard errors. Statistical significance was determined by one‐way ANOVA with Tukey's multiple comparison test (*p < 0.05; **p < 0.01; ***p < 0.001; ****p < 0.0001) using GraphPad Prism v10 (GraphPad Software, Boston, MA, USA).

## Conflicts of Interest

The authors declare no conflicts of interest.

## Supporting information




**Supporting File 1**: adma74822‐sup‐0001‐SuppMat.docx.

**Supporting File 2**: adma74822‐sup‐0002‐MovieS1‐S4.zip.

## Data Availability

The data that support the findings of this study are available from the corresponding author upon reasonable request.
