# Supplementary material for: Upscaling the Fabrication of Rod‐Shaped Microgels: A Microfluidic Method Combining Step‐Emulsification With Consecutive Droplet Confinement in Parallelized Microchannels
Source: Adv Mater. 2026 Aug 28;38(59):e74822. doi: 10.1002/adma.74822 (PMC13689903; doi:10.1002/adma.74822)
Supplement: Supplementary file 1 [file ADMA-38-e74822-s002.docx]

**Supporting Information**

**Upscaling the fabrication of rod-shaped microgels: a microfluidic method combining step-emulsification with consecutive droplet confinement in parallelized microchannels**

Matthias Mork^a^, Ninon Möhl^a^, Greta Romahn, Laura Klasen, Cédric Bergerbit, Laura De Laporte*

1. **Microfluidic systems to fabricate rod-shaped microgels**

**Table S1.** **Overview of selected microfluidic system for fabricating rod-shaped microgels.** Comparison of the production of biocompatible rod-shaped microgels for tissue engineering applications, using different microfluidic methods (SE+DC: Step-emulsification and droplet distribution and confinement, FFM: Flow-focusing microfluidics, CJP: compartmentalized jet polymerization, DMT: droplet-based microfluidic tubing). The maximum flow rate Max. Q_disp_ is related to the minimum possible width.

| **Paper** | **Method** | **Material** | **Min. width [µm]** | **Max. *Q_disp._*[mL/h]** |
| --- | --- | --- | --- | --- |
| This work | SE+DC | PEG-DA 700 or  sPEG-NB + sPEG- SH | 50 | 3 |
| Rodriguez-Rivera et al. 2025 | FFM | Norbornene-HA | 107  68* | 0.15  0.4 |
| Qazi et al. 2022 | FFM | Norbornene-HA | 100 | 0.15 |
| Rommel et al. 2022 | FFM | sPEG-Acrylate | 128 | 0.03 |
| Kittel et al. 2023 | FFM | sPEG-Acrylate + PEG-DA 700 (blends) | 80 ** | 0.035 |
| Guerzoni et al. 2019 | FFM | sPEG-VS + sPEG-SH | 80 | 0.06 |
| Krüger et al. 2019 | FFM | PEG-DA 700 | 80 | 0.13 |
| Krüger et al. 2019 | CJP | PEG-DA 700 | 8 | 0.01 |
| Möhl et al. 2025 | CJP | PEG-DA 700 | 3 | 0.005 |
| Ma et al. 2017 | DMT | GelMa or Matrigel | 50 | 0.25 |
| Lee et al. 2024 | Parallelized FFM | PEG-DA 575 | 63 | 0.8*** |

*: Fabricated under flow perturbation according to study, resulting in un-uniform microgel geometries

**: channel width; microgel size not specified.

***: Total flow rate, as *Q_disp._* was not explicitly specified.

2. **Previous Microfluidic Master Prototypes**

During the development of the microfluidic master, two different prototypes were tested, having different geometrical features. Both prototypes featured a SE section for droplet generation, but included different geometries to distribute the droplets into parallelized microchannels (50 x 50 µm²). We compared directly connecting an array of parallelized microchannels to the SE section versus implementing a droplet distribution section, which was achieved by splitting the main channel gradually into individual microchannels. While a direct connection between the SE section and microchannel array resulted in an irregular droplet flow velocity within the parallelized microchannels, the implementation of a droplet distribution section resulted in a more uniform droplet flow velocity. Additionally, we compared a steep (90° angle) versus an inclined height-width transition for droplet elongation. The inclined geometry achieved a higher flow stability, reducing droplet coalescence and accumulation of droplets or residual air in the space above the entrance to the confinement channel. In the second prototype, we also implemented triangular geometries within the channel to induce droplet splitting, as previously reported ^[1]^. By implementing this, the theoretical production rate is further increased, as a single initial droplet will yield two rod-shaped microgels. However, we observed an increased heterogeneity of the microgel rod length due to asymmetric droplet splitting. We attribute this effect to slight pressure differences in the individual channels, causing the droplet to asymmetrically expand into the parallel microchannels. Consequently, we removed the droplet splitting section from our finalized microfluidic chip design.

**Figure S1.** **Overview of the first and second microfluidic chip prototype designs.** A) Top view 2D representation of the first microfluidic chip design, featuring a step-emulsification (SE) section and a confinement section, composed of nine parallelized microchannels. B) Microscope image of the first chip design during operation. The generated spherical droplets are squeezed into the confinement channels, creating elongated droplets. The first chip design features a step height-width transition at a 90° angle between the oil reservoir and the confinement channels. C) 2D representation of the second chip design, including a distribution and splitting section. D) Microscope image of the second microfluidic chip design showing the droplet distribution and splitting. The second chip design features an inclined height-width transition. The inset displays a schematic of symmetric and asymmetric droplet splitting. E) Exemplary comparison of the uniformity of droplet flow between two video frames in the first and second chip design. I: The left image displays the first chip design at the first frame with selected droplets highlighted in blue. In the second frame (right), the blue color indicates the initial droplet position, and red highlights the droplet position in the second frame. The black bars in between the two droplet positions visualize the covered distance (Δd). II displays the same for the second chip design. F) Microscope images displaying the production of rod-shaped microgels in the first (I) and second (II) chip design. In all microscope images displaying the first and second prototype designs, the flow rates were set to *Q_cont._*= 0.3 mL/h, *Q_disp._*= 0.1 mL/h and *Q_cont._*= 0.6 mL/h, *Q_disp._*= 0.3 mL/h, respectively. All scale bars represent 200 µm.

**Figure S2. Optimized microfluidic chip design with characteristic dimensions.** All values are displayed in µm.

**Figure S3. Optimized microfluidic chip design during operation.** A) Microscope images showing the droplet generation in the SE section and droplet elongation in microchannels at different applied flow rates. B) Droplet elongation at different continuous phase flow rates while the dispersed phase flow rate was kept constant at 1 mL/h. All scale bars are equal to 250 µm.

3. **Influence of the nozzle geometry on the resulting droplet sizes**

We additionally fabricated microfluidic masters with identical dimensions, but a reduced nozzle channel height of 8 µm and 12 µm in the step-emulsification section. A reduction in nozzle height decreases the total available volume per nozzle channel, directly impacting the resulting droplet size. As shown in Figure 2C, for a nozzle height of 17 µm, we measured a droplet diameter of approximately 102.32 ± 4.63 µm, at a flow rate combination of *Q_disp. :_ Q_cont._*= 1:2 mL/h. Maintaining the same flow rate resulted in droplet diameters of 75.75 ± 1.85 µm and 98. 25 ± 5.38 µm for the 8 µm and 12 µm nozzle height, respectively (**Figure S4 A+C**). Consequently, the elongated droplet lengths reduced to 136. 08 ± 10.94 µm, and 238.95 ± 34.96 µm, respectively (**Figure S4 A+D**). After this experiment, we adjusted the flow rates for each device to match the flow rate per nozzle volume in each device to reduce potential alterations of the droplet sizes due differences in flow per nozzle, which has shown to influence the resulting droplet sizes (**Figure 2C**). At the adjusted flow rate conditions, the droplet diameters further decreased to 59.57 ± 2.19 µm and 87. 44 ± 3.71 µm, for the 8 µm and 12 µm nozzle height, respectively (**Figure S4 B+C**). The elongated droplet lengths were reduced to 75.58 ± 6.23 µm and 192.78 ± 29.74 µm, respectively (**Figure S4 A+D**).

**Figure S4. Tuning of the droplet sizes by variation of the nozzle channel height.** A) Microscopy images showing microfluidic devices with a nozzle channel height of 12 µm and 8 µm during operation at flow rates of 1 mL/h for the dispersed phase and 2 mL/h for the continuous phase. B) Microscopy images of the same microfluidic devices at a matched flow rate per nozzle volume, calculated based on the difference in nozzle volume. C+D) Influence of the applied flow rates on the initial spherical droplet diameter (C) and the length (D) of the elongated droplet within the confinement microchannel. n = 50 spherical droplets and elongated droplets. Statistical significance was determined by one-way ANOVA with Tukey’s multiple comparison test (*p < 0.05; **p < 0.01; ***p < 0.001; ****p < 0.0001).

4. **UV dose calculation**

The intensity of the diode used for on-chip gelation was measured on a Thorlabs photometer (PM400) equipped with a Thorlabs detector (Model S170C). The diode provided a controllable UV intensity up to a maximum value of 1353 mW/cm^2^ at a spot size of ≈ 6 mm.

The flow velocity *U* (m/s) was calculated by the ratio of the applied total flow rate Q_total_ (mL/h) and the cross-section area of the microchannels *A_cross_ (*cm^2^*)*. As in the parallelized device, the flow velocity is distributed over multiple microchannels, the number of microchannels (*N*) is included as an additional factor in the calculation, as displayed in equation (1).

$$U=\frac{Q_{total}}{A_{cross.}*N}$$

*(1)*

The residence time t (s) within the UV spot was calculated by the ratio of the channel length *L* within the UV irradiation field (parallelized device = 5.5mm, single-channel device = 6mm) and the flow velocity *U*.

$$t=\frac{L}{U}$$

*(2)*

The UV dose (mJ/cm^2^) per microchannel was calculated as the product of the UV intensity *I* (mW/cm^2^) and the residence time *t*.

$$UV Dose=t\cdot I$$

*(3)*

**Figure S5. Calculated UV dose per individual droplet confinement channel in dependence of the applied flow rate.** In our parallelized microchannel device (black circles) the UV dose is higher if compared to an equally sized single channel device (white circles). The vertical dashed line indicates the minimum determined UV dose of 31 mJ/cm^2^ to produce rod-shaped microgels at 30% (w/w) PEG-DA. Depicted values were calculated by considering a constant UV intensity of 1353 mW/cm^2^ for both devices and a length of irradiation area of 5.5 mm and 6.0 mm, respectively.

**Figure S6. Fouling assessment in the microfluidic device after a continuous production of rod-shaped microgels for over 200 min.** The top row indicates selected regions of the microfluidic chip design before use. The bottom row displays the same regions after continuous microgel rod production.

**Figure S7. PEG-DA microgel rod production at lower PEG-DA concentration.** Analysis of the applied UV dose required to fabricate stable microgel-rods (15% (w/w) PEG-DA 700) at a total flow rate of 3 mL/h. Scale bar equal to 200 µm.

**Figure S8. Mechanical properties of bulk PEG-DA and PEG-Norbornene/SH hydrogels obtained through rotational rheology.** Bars represent the storage modulus, while the points correspond to the gelation time. Data points are means ± standard errors. Statistical significance was determined by one-way ANOVA with Tukey’s multiple comparison test (*p < 0.05; **p < 0.01; ***p < 0.001; ****p < 0.0001).

**Figure S9.** **Microscope images showing microgel rod production based on 5 % (w/v) sPEG-SH/NB.** The microgels were produced at different flow rates and subjected to different UV intensities. The scale bar = 200 μm and is representative for all images in this series.

**Figure S10.** **Production of microgels rods using 3 % (w/v) of sPEG-SH/sPEG-NB.** The microgels were irradiated at 351 mW/cm^2^ at flow rates of 1 mL/h for the dispersed phase and 2 mL/h for the continuous phase.

**Figure S11.** **Cell experiments with microgel rods and C2C12 mouse muscle cells.** Left row shows a condition of only cells. The middle row shows the interaction of cells and PEG-DA 30% (w/w) microgels without RGD-modification. The right row shows cell interaction on sPEG-SH/NB 5% (w/v) microgel rods without RGD-modification. Initial starting cell amount was 20,000 cells per condition. Scale bars all represent 100 µm.

**Supplementary Movies**

**Movie S1. Optimized microfluidic chip design during operation showing the droplet generation and elongation at different applied flow rates.**

**Movie S2: Continuous production of microgel rods based on 30% (w/w) PEG-DA 700, fabricated over a total time of 200 minutes at flow rates of Q_cont._= 2 mL/h and Q_disp._= 1 mL/h.**

**Movie S3: Continuous production of microgel rods based on 5% (w/v) sPEG-SH/sPEG-NB, fabricated over a total time of 100 minutes at flow rates of Q_cont._= 2 mL/h and Q_disp._= 1 mL/h.**

**Movie S4: Analysis of the applied UV dose required to fabricate stable microgel-rods (5% (w/v) sPEG-SH/NB) at flow rates of Q_cont._= 2 mL/h and Q_disp._= 1 mL/h.**

**References**

[1]	A. R. Abate, D. A. Weitz, *Lab Chip* **2011**, *11*, 1911.
